# Integrating polyspikes and excitability markers for epileptic network identification

**DOI:** 10.3389/fnetp.2026.1966953

**Published:** 2026-10-14

**Authors:** James E. Niemeyer, Benjamin L. Barnes, Nitish Seenarine, Meritxell Sainz Barrionuevo, Theodore H. Schwartz

**Affiliations:** 1 Department of Neurosurgery, Weill Cornell Medicine, New York, NY, United States; 2 Department of Surgery, SUNY Downstate Health Sciences University, New York, NY, United States; 3 Northwestern University, Evanston, IL, United States; 4 Department of Neurosurgery, Icahn School of Medicine at Mount Sinai Hospital, New York, NY, United States

**Keywords:** adaptive neurostimulation, brain mapping, control theory, epileptic network, network mapping, drug resistant epileptic network, brain network identification

## Abstract

Epilepsy is increasingly recognized as a disorder of distributed brain networks rather than a disease arising from a single seizure focus. Although clinical practice remains centered on localization of the seizure onset zone (SOZ), persistent seizures following apparently successful resection suggest that epileptogenic activity is often supported by broader networks of interconnected regions. A major challenge for both surgical and neuromodulatory treatment is identifying not only where seizures occur, but also which connected brain structures are components of a network that may underlie the generation of seizures. Here, we propose network excitability mapping as a translational framework for characterizing epileptic networks. In this view, epileptic networks can be described along two complementary dimensions: 1) local excitability and 2) network recruitment. We highlight evidence that the aperiodic (1/f) slope of field-potential recordings provides a mechanistically grounded measure of local excitability, while polyspike structure can reflect the degree to which epileptiform activity recruits distributed network nodes. Recent studies, and preliminary data presented here, combining electrophysiology, calcium imaging, and computational modeling suggest that these measures capture distinct but related aspects of epileptic network function. Together, these approaches support a shift from SOZ-based paradigms toward functional mapping of epileptic networks. We argue that combining measures of excitability and recruitment may enable construction of individualized excitability maps capable of informing surgical planning, optimizing neurostimulation, and improving seizure control. Establishing translational biomarkers of network engagement will be critical for advancing precision therapies in epilepsy.

## Introduction

Drug-resistant epilepsy (DRE) is a major clinical challenge that afflicts about 1 in 3 patients with epilepsy (Sultana et al., 2021). Traditional approaches focus on identifying and removing a seizure onset zone (SOZ), but many patients continue to experience seizures, even despite apparently successful localization and resection (Barba et al., 2022; Englot et al., 2012). This indicates that, in many cases, epilepsy is not a disorder of a single focus but rather involves a complex interaction of interconnected brain sites. Thus, a problem for improving patient outcomes is that clinical strategies often struggle to capture this network complexity.

Identifying an SOZ is itself difficult, and even when successful, resection does not guarantee seizure freedom (Englot et al., 2012; Englot and Chang, 2014; Avigdor et al., 2026). When this occurs, it is likely because seizures can arise from multiple regions, emerge from latent secondary sites, persist through network reorganization after surgery, or combinations of these etiologies (Blumenfeld et al., 2009; Burns et al., 2014; Choy et al., 2021; Fan et al., 2022; Spencer, 2002). This suggests that the SOZ may not represent the generator of seizures, but rather one vulnerable node within a broader network capable of initiating or supporting pathological hyperactivity and synchrony. Thus, localization of a putative SOZ only offers a partial picture of epileptic function, as was seminally highlighted by Susan Spencer in 2002 (Spencer, 2002; Zaveri et al., 2026). In a *seizure network*, seizures emerge from interactions among distributed regions, each with distinct levels of excitability, connectivity, and tendency toward hypersynchronous activity. In this framework, the key determinant of seizure generation is not only where activity begins, but how pathological excitation propagates through a network, and which nodes are capable of amplifying or constraining that activity. However, there are currently few ways to directly measure or map this network-level excitability in either patients or experimental animal models. To bridge this gap, we argue researchers should integrate mechanistic insights from preclinical models with quantifiable signals in human electrophysiology. Animal models, particularly mice, have made it possible to dissect the circuit-level dynamics of seizure propagation and recruitment (Choy et al., 2021; Niemeyer et al., 2025b; Brodovskaya et al., 2022; Brodovskaya and Kapur, 2019; Tao et al., 2026; Brodovskaya et al., 2021). However, few of these insights have so-far been leveraged into clinically useful biomarkers in humans (Sin et al., 2022). Identifying electrophysiologic signatures that reflect network engagement, and that are seen in human patients, would provide a link between basic neuroscience and clinical practice, and could provide actionable next steps to improve patient outcomes.

In this article, we describe this problem of network identification and highlight a combination of electrographic features that may offer new treatment directions. Patterns of polyspike activity can reflect the degree to which seizures engage distributed networks rather than remaining localized, while the recently described 1/f aperiodic slope offers a useful index of excitability. We first provide background on epileptic and seizure network formation and then highlight combined electrophysiological and calcium imaging evidence from mouse models demonstrating that specific spike waveforms can provide information about network recruitment. This suggests that electrographic features observable in clinical recordings may contain information not only about where epileptiform activity occurs, but also about how broadly it engages seizure networks. Further, we highlight how mechanistic studies in animal models are essential for defining how network excitability manifests across epileptic circuits and for identifying biomarkers that can ultimately be translated into human studies and stimulation-based therapeutic strategies.

## Network mapping in human patients

### Computational models

Supported by a clear role for networks in both epileptogenesis and treatment, theoretical and computational scientists have already developed models that treat epilepsy as a disorder of networks (Ren et al., 2019; van Diessen et al., 2013; Ashourvan et al., 2020; Chari et al., 2022). Graph theory has been especially influential by representing the brain as a set of nodes joined by connections (or edges), essentially connecting lines of varying strengths or thicknesses. This lets neuroscientists quantify not only whether connections are strong or weak, but properties such as how central a node is and how readily activity travels between regions, at scales ranging from single neurons to the whole brain. The approach can, for example, identify highly connected “hub” nodes that exert disproportionate influence over how seizures spread (van Diessen et al., 2013). Critically, when applied to human epilepsy, such network measures have predicted surgical and neurostimulation outcomes more accurately than anatomical localization of the seizure onset zone alone (Fan et al., 2022; Khambhati et al., 2021).

A second computational approach goes beyond describing the brain as a generic network. Instead of representing the brain as abstract nodes and connections, the virtual epileptic patient (VEP) provides a detailed, patient-specific model (An et al., 2019). Brain regions reconstructed from the patient’s MRI serve as nodes, white matter pathways provide the connections, and each region receives a mathematical model of seizure activity. This lets the model simulate how a seizure spreads through that individual’s network, estimate the epileptogenic zone, and even run “virtual surgery” to test which resections would best control seizures (Dollomaja et al., 2025; Makhalova et al., 2022; Wang et al., 2024). VEP has been validated retrospectively in 53 patients, where the completeness of resection of the VEP-predicted network tracked outcome: 91% of it was removed in patients who became seizure-free, versus 55% in those whose seizures persisted (Makhalova et al., 2022), and a prospective trial is now underway (NCT03643016) (Dollomaja et al., 2025).

### Limits of inference

Despite the promise of graph-theoretic and virtual patient models, these approaches largely infer network organization from passively recorded neural activity. While they can identify candidate hubs, epileptogenic regions, and pathways of seizure propagation, they do not directly test how specific network elements contribute to seizure generation. To address this limitation, several groups have used electrical stimulation as an active probe of epileptic networks. Single-pulse stimulation and corticocortical evoked potentials (CCEPs) can reveal effective connectivity (Miller et al., 2021), uncover tipping points at seizure onset not apparent during spontaneous activity (Graham et al., 2023), and in some cases help localize seizure-generating regions (Oderiz et al., 2019; Sivaraju et al., 2024). Together, these studies provide important evidence that epilepsy often reflects dysfunction of distributed networks rather than a single isolated focus. However, stimulation-based approaches have significant practical limitations. They require invasive monitoring, provide only sparse sampling of large-scale networks, and are difficult to apply systematically across all patients.

Therefore, there is a critical need for biomarkers that can be extracted from routine electrophysiological recordings and that more directly capture the mechanisms underlying seizure generation and network recruitment. Below we describe how metrics such as the aperiodic slope of power spectral activity at different sampled brain regions, and the occurrence of polyspikes, can provide complementary measures of local excitability and network involvement. These features offer a new approach for identifying 1) the excitability across a brain network and 2) when epilepsy arises from, or engages, distributed networks rather than a single focal source.

## Current markers localize epileptogenic tissue but may not index excitability

Clinically, the SOZ is identified from electrographic features related to seizure onset or interictal periods. Seizure onset detection is limited by the short temporal window (often on the order of days) to sample seizures and their unpredictability. Thus, interictal activity is a more abundant and reliable measure. Features like interictal spikes and high-frequency oscillations (HFOs) have long been used to delineate tissue for resection (Lagarde et al., 2019; Grinenko et al., 2018; Jobst et al., 2020) and are valuable, but each essentially identifies tissue that behaves abnormally and is associated with seizures. Neither of these features directly report how the excitability of a given node, nor whether this tissue is a component of a broader epileptic network. These limitations may underly the cases of surgical failure: In one clinical study (Holmes et al., 2000), researchers found that surgical resection in patients with multifocal interictal spikes experienced roughly 33% seizure freedom compared with 77% seizure freedom for those with localized interictal spikes. In other retrospective studies, when interictal spikes detected by electrocorticography are used to guide surgical resection, about 20%–40% of patients continued to experience seizures (Guo et al., 2024; Demuru et al., 2020; Goel et al., 2023). Similarly, in one study that used intracortical encephalography (iEEG) to monitor both seizures and interictal spiking, it was found that the seizure markers were significantly more effective at identifying ictogenic sites (Makhalova et al., 2023), further supporting the view that simply identifying interictal spiking sites does not provide an optimal picture of the seizure generating brain site. Meanwhile, HFOs were long believed to provide a strong indication of ictogenic brain sites, but this has been increasingly questioned (Jacobs et al., 2018; Wang et al., 2022; Roehri et al., 2018). In a recent randomized controlled study it was found that HFOs do not outperform conventional interictal spikes in identifying these sites. In fact, seizure freedom was 23% lower in the HFO-guided arm of the study compared with spikes (Zweiphenning et al., 2022). In one large study of 341 patients who underwent SEEG followed by targeted resection, about 45% continued to experience seizures at 1 year follow up (and 60% at 5 years follow up). Notably, this study highlighted that of an original 536 patients who underwent SEEG, it was only the 341 patients with “identified” seizure generating sites that chose to pursue resection, demonstrating the perceived significance of finding the “onset” zone.

Overall, studies like these support the utility of current markers of seizure onset and interictal epileptiform activity. However, clinical outcomes show that identifying epileptic tissue alone is often insufficient. Although many patients with drug-resistant epilepsy benefit from surgical treatment directed at the SOZ, seizure freedom is far from guaranteed. This gap suggests that the regions generating detectable epileptiform activity may not always be the same regions that possess the greatest excitability or play the most important role in initiating and recruiting the broader epileptic network. Developing biomarkers that capture these properties could therefore improve our ability to identify the key drivers of seizure generation.

## Aperiodic slope as an excitability index

One recently popularized metric of excitability that could meet this need is the aperiodic, or 1/frequency (1/f), component (Donoghue et al., 2020) of the field-potential power spectrum recorded by intra- or extra-cranial electrodes. Plotted on logarithmic axes, spectral power falls with frequency along an approximately straight line, and the steepness of that line (the aperiodic exponent with a linear fit) reflects the local balance of synaptic currents. The interpretation of the aperiodic slope value as a sign of local excitability is supported by several lines of research. Computational modeling first linked variations in aperiodic slope to changes in excitation-inhibition balance (Gao et al., 2017), and subsequent studies have associated regional differences in slope with excitability-related gene expression in epilepsy (Duma et al., 2024). Causal manipulations in animal models further support its physiological relevance, with both optogenetic and pharmacological modulation of inhibitory circuits producing predictable changes in slope (Wang et al., 2026). While the relative importance of excitatory and inhibitory activity in the aperiodic slope is still being understood, the aperiodic slope does provide an unambiguous marker of the relative amount of high frequency neural activity. It thus serves as a proxy for local excitability, extractable from data already passively collected using established spectral-parameterization methods (Donoghue et al., 2020).

In humans, the aperiodic exponent is associated with epileptic tissue (Kozma et al., 2024; Kundu et al., 2023), however, it is not currently known whether the aperiodic slope is causally linked to seizure generation. One way to test this is to identify if the aperiodic slope changes before and after electric stimulation with a responsive neurostimulation system (RNS); these systems are designed to first identify a potential oncoming seizure and then apply stimulation meant to halt these events. To examine this, in a small preliminary experiment, we analyzed data from patients undergoing RNS treatment. We used a publicly available RNS and iEEG data set from Feng et al. (2026) and analyzed the aperiodic slope one minute before (early), 20 s before (middle), and 20 s after the period of neurostimulation (post-stimulation). We found that, across all 8 patients, the aperiodic slope was significantly higher immediately prior to stimulation, and significantly lower following electrical stimulation (Figure 1). Aperiodic slope values were averaged within subjects over recordings and differences among epochs (early, middle, post-stim) were evaluated using a repeated-measures test, which showed significant differences across periods for both EEGs (Friedman χ^2^ (2) = 14.25, p = 0.0008). The post-stim period exhibited a significantly decreased change in slope following stimulation compared to immediately prior to stimulation (Figure 1B). These results provide suggestive (non-experimental) evidence that the aperiodic slope of iEEG signal is a meaningful indicator of ictogenesis that is changed (lowered) following electrical stimulation treatment.

**FIGURE 1:**
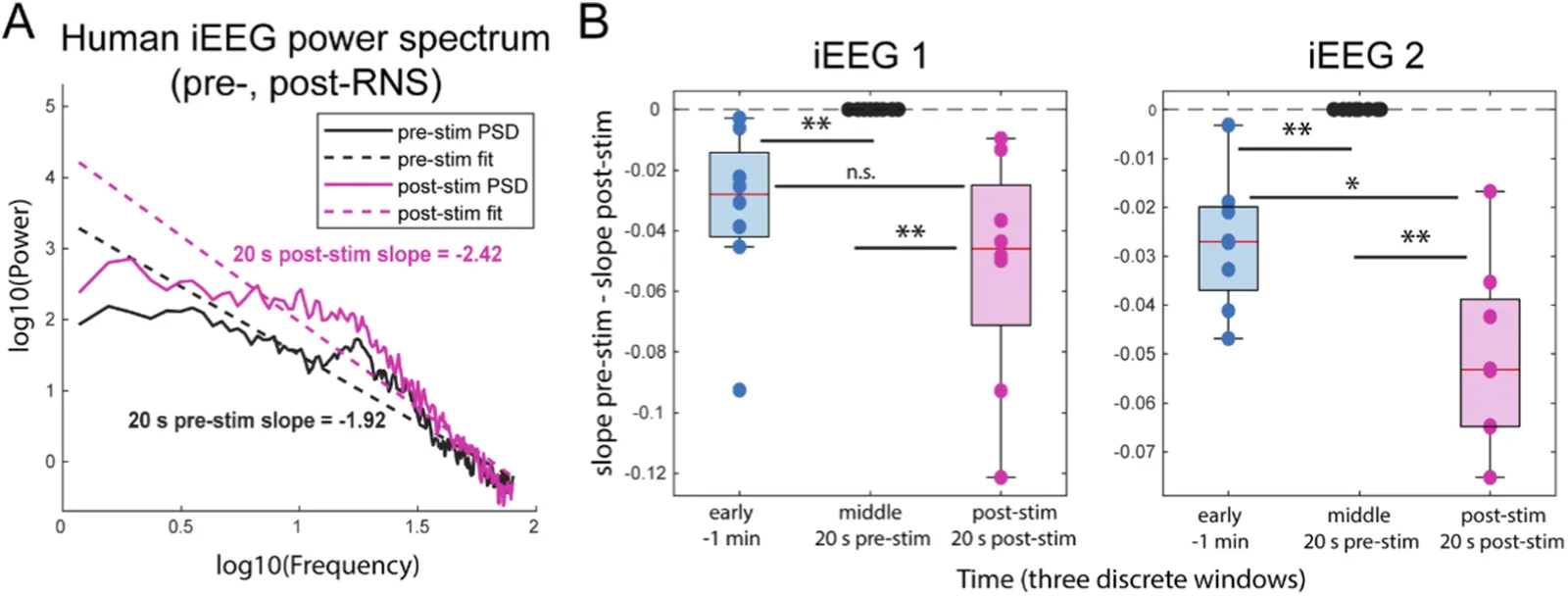
Aperiodic slope is significantly altered following focal responsive neurostimulation. **(A)** the power spectrum of iEEG signal before and after a bout of RNS stimulation (single trial) shows a change in relative contributions of lower vs. higher frequencies. **(B)** Measuring electrographic data from immediately prior to and following RNS stimulation, we find that the aperiodic slope is significantly higher immediately prior to stimulation and significantly lower immediately after stimulation (*p <0 .05, **p <0 .01, Wilcoxon signed-rank test). Across linear fits, the median R^2^ values ranged from 0.91 to 0.94 across all analysis windows and EEG channels, indicating a single exponent accounted for the large majority of the spectral variance.

During epilepsy monitoring, in which clinicians seek to identify icotgenic zones for resection or stimulation treatment, it is common for patients to be implanted with many iEEG recording electrodes. By calculating the aperiodic slope, each of these contacts could provide a unique spatiotemporal excitability signature. We argue that, applied across intracranial contacts and recorded over various brain states (e.g., sleep, wake, active task), this information could produce a node-by-node map of excitability, which, based on the above evidence, is likely to correlate with the true underlying epileptic brain network. Such a map could provide actionable extra-focal targets (Tao et al., 2026; Piper et al., 2022) for resection and neurostimulation treatments that currently focus solely on the SOZ. While the simple method of fitting the power spectrum slope has benefits, there are also significant limitations, particularly an assumption of some stationarity in the time window for analysis (Gorshkov and Ombao, 2024), but also other considerations and areas for future exploration which we describe below.

## Polyspikes as indicators of network involvement in epilepsy

Mapping local excitability across distributed iEEG contacts is likely useful towards identifying a putative epileptic network, but characterizing this network also requires knowing whether epileptiform activity is co-occurring or co-generating across a set of connected brain sites. We propose that an electrographic feature may speak directly to that question: the polyspike. Defined as a sequence of two or more consecutive spikes (subspikes), often followed by a slow wave, polyspikes are an established feature of the epileptic EEG, classically associated with genetic generalized epilepsies such as juvenile myoclonic epilepsy (Dhanuka et al., 2001; So et al., 1998; Sadleir et al., 2009; Serafini et al., 2013). However, their significance in focal epilepsy, and the generators that produce them, remain understudied.

There are two general explanations for the underlying generation of polyspikes. One possibility is that rapid recruitment of two or more populations of neurons around the electrode can co-occur with slight temporal delays that create an appearance of a multiple component spikes riding upon a single wave in the local electric field recording. A second possibility is that each component spike reflects recurrent excitation returning through partially distinct pathways within a distributed seizure network. As additional nodes become recruited, activity repeatedly re-enters a focal recording site through reciprocating or reverberating circuit interactions, generating the characteristic clustered appearance of polyspike discharges. In this view, polyspikes emerge not merely as markers of local hyperexcitability, but as signatures of communication among interconnected network elements.

We have recently analyzed epileptiform activity, which supports the second possibility (Figure 2). Using widefield calcium imaging across a multi-node bilateral neocortical network (ipsi- and contra-lateral somatosensory and secondary motor cortices) during acute chemoconvulsant (4-Aminopyridine) seizures, a preparation in which the spatial extent of recruitment can be measured directly (Niemeyer et al., 2025a; Niemeyer et al., 2025b), polyspike structure tracks network involvement (Figures 2A,B). Polyspike duration is positively correlated with the cortical area recruited (*r* = 0.53, p < 0.00001; n = 171 polyspikes across six mice), and more complex polyspikes, composed of multiple subspikes, recruit network nodes more reliably than isolated spikes. The relationship is not a linearly simple “three subspikes, three network nodes”, but rather recruitment is graded and often extends beyond the S1-M2 network. However, the internal structure of a focal event indexes how broadly the seizure has spread, a link that becomes visible only when imaging provides the ground-truth recruitment that electrographic traces alone cannot. However, we also note that our methods cannot completely prove that focal effects are not involved in polyspike structure, an issue that may require fast voltage imaging in future experiments (Villette et al., 2019; Piatkevich et al., 2019). With a better understanding how polyspike structure correlates with contra- and ipsilateral network recruitment, it is likely that these electrographic features can be used to provide a fast readout of whether a broad brain network is involved in a particular subject’s epilepsy.

**FIGURE 2:**
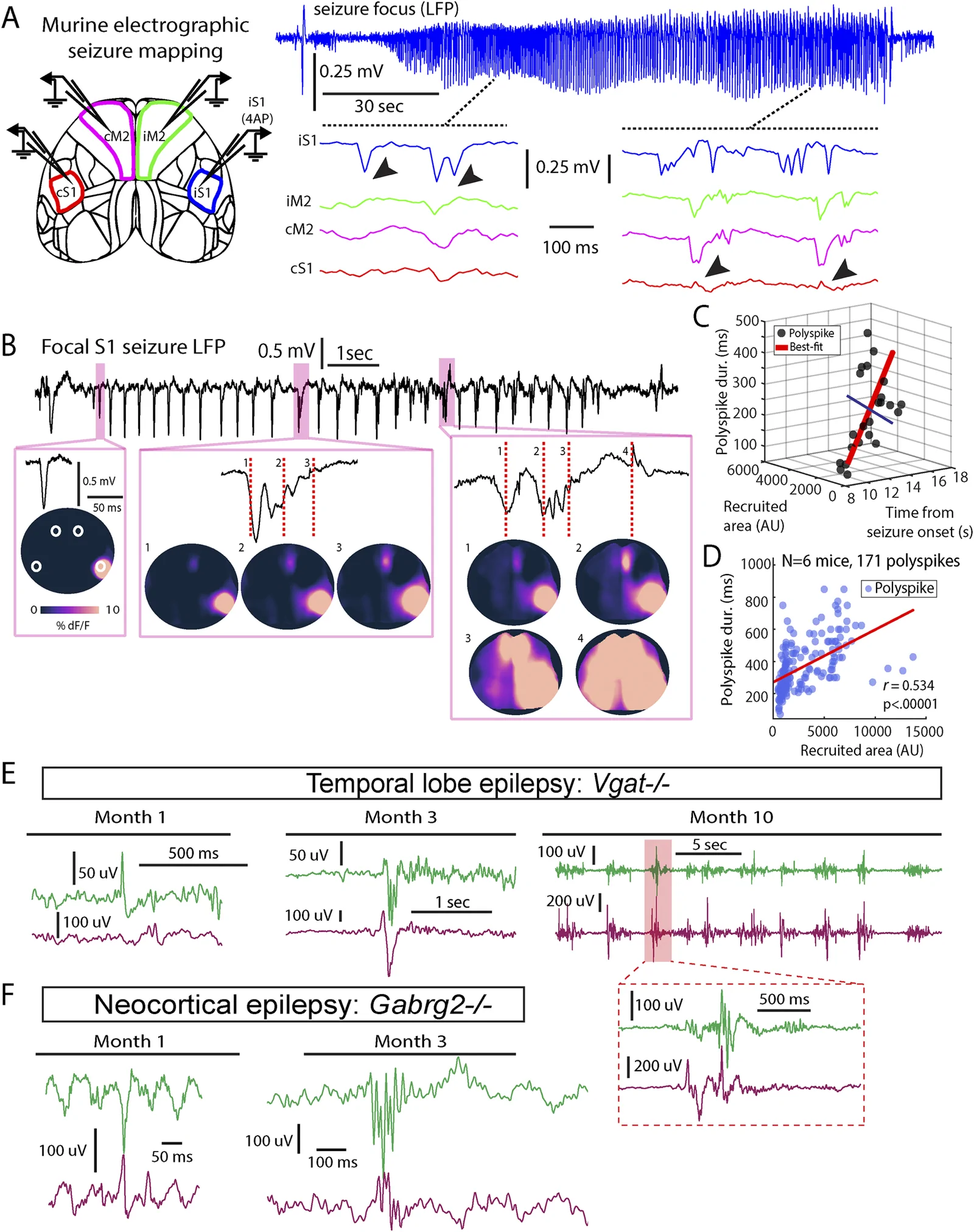
Polyspikes identify network recruitment and develop throughout epileptogenesis. **(A)** In a pilot experiment, electrodes were inserted into four anatomically connected brain sites comprised of primary somatosensory cortex (S1) and secondary motor cortex (M2); ipsi (i) and contralateral (c) components are connected by the corpus callosum. During a seizure that was initiated in S1, using the chemoconvulsant 4-Aminopyridine, polyspikes increase in size over time, and events in one region correspond to recruitment of events in other regions (arrows). **(B)** Using widefield calcium (GCaMP6f) imaging at 60 Hz, this experiment shows a correlation between electrographic (black traces) polyspike size and the recruitment of the anatomically connected sites (white circles) in the mouse dorsal cortex (Niemeyer et al., 2025a). **(C)** The polyspike duration was correlated with the brain area recruited for this event, as well as **(D)** multiple seizure events in different mice. **(E)** Using a focally injected adeno-associated virus (AAV) in the temporal lobe (dorsal hippocampus) to delete the inhibition-related *Vgat* gene, we find that EEG polyspikes increase in size over a 10-month period of epileptogenesis. Green traces are EEG above the AAV injection (seizure focus), while magenta traces are ipsilateral but 5 mm distant. **(F)** This was also seen in a 3-month period in a pilot experiment that deleted a different gene (*Gabrg2*) in the neocortex. These recordings were performed with skull EEG during 24-h monitoring with a Pinnacle Video-EEG system at 400 Hz. Polyspikes were identified with a custom MATLAB-based semi-automated detection algorithm and visually confirmed.

Beyond acute seizure recruitment, we have also examined EEG data from two longitudinal focal epilepsy models, finding evidence that polyspikes evolve, broaden, and increase in frequency during multi-month epileptogenesis. These models are created by using *Vgat* ^*fl/fL*^ (Jackson Lab #012897)or *Gabrg2* ^*fl/fl*^ (Jackson Lab #016830) mice followed by AAV-mediated Cre (AAV9. CaMKIIa.Cre, Addgene) expression in either the hippocampus (*Vgat*) or frontal secondary motor cortex (*Gabrg2*). In this paradigm, Cre is expressed in CaMKIIa + neurons leading to conditional loss of *Vgat* or *Gabrg2*, both genes important in neural inhibition. In both hippocampal *Vgat* deletion and cortical *Gabrg2* deletion animals, polyspike duration and complexity increased over time as epileptiform activity developed (Figures 2E,F). This initial finding suggests the possibility that polyspikes are not only markers of momentary network recruitment but may also indicate progressive network reorganization or strengthening occurring over the course of disease development.

Because polyspikes are readily measurable in conventional electrophysiologic recordings, they represent an attractive candidate biomarker for detecting seizure networks in humans. Importantly, animal models provide a unique opportunity to establish the biological significance of these events because simultaneous imaging can directly reveal the extent of network recruitment that underlies individual electrographic waveforms. Thus, polyspikes may complement established clinical markers such as interictal spikes and high-frequency oscillations. Whereas these markers help identify tissue associated with epilepsy, polyspike structure may provide information about how strongly a given region participates in the distributed seizure network. Future studies in patients will be required to determine whether the same relationships observed in experimental models are preserved in human epilepsy and whether polyspikes can identify hub-like regions that disproportionately contribute to seizure spread.

## Clinical translation and future directions

We propose that local excitability and network recruitment represent separate but complementary dimensions of epileptic networks. Measures such as the aperiodic slope may provide information about a node’s propensity to generate pathological activity, whereas polyspike structure may reflect the extent to which that activity engages other network components. Together, these dimensions begin to define an excitability map that extends beyond localization of individual epileptogenic sites and toward a more complete description of seizure-network dynamics.

Building the node-by-node map of excitability described above from the aperiodic exponent of resting iEEG may reveal which nodes are excitation-dominant amplifiers worth targeting and which are inhibition-dominant and best preserved. An important future goal will be to determine whether network biomarkers can identify highly influential hub or amplifier nodes, as the present work does not establish directionality between recruited brain sites (something which may be determined by analyses incorporating transfer entropy, generalized synchronization metrics, or stimulation-based probing). Computational and graph-theoretic studies have suggested that certain nodes exert disproportionate influence over seizure spread and treatment response. If polyspikes preferentially emerge from such regions, they may provide a practical electrophysiological marker of network influence that complements anatomical and connectivity-based approaches. Identifying these amplifier nodes could aid both surgical planning and selection of stimulation targets. Because it draws on recordings already gathered at presurgical evaluation, such a map reframes the network in functional, not merely anatomical, terms.

Meanwhile, electrographic waveform features, like densely packed subspikes, may also provide a practical biomarker for evaluating neuromodulation strategies. If polyspikes indeed reflect network recruitment, then successful stimulation paradigms may reduce their frequency, complexity, duration, or associated network engagement. Because these features can be measured longitudinally from implanted recording systems, they may serve as an early indicator of network-level therapeutic effects before changes in seizure frequency become apparent. Future experimental and clinical studies should determine whether stimulation-induced alterations in polyspike activity predict long-term treatment response.

These features together provide a noteworthy opportunity for machine learning. Any responsive or deep brain stimulation device that records activity could track how that activity changes under different stimulation parameters. This would be particularly important when a device may be failing to abort seizures yet still be reshaping the network: certain parameters might alter the structure of interictal polyspikes long before seizure frequency changes, an early signature of chronic network reorganization through which these therapies are thought to act (Rao and Rolston, 2023). The fine structure of these events like subspike count, prominence, and the amount of co-recruitment they predict is the kind of high-dimensional, network-sensitive feature machine learning is suited for. Coupled to an excitability map and a closed-loop device, such biomarkers could let parameters be chosen and continuously refined on the basis of network engagement rather than seizure counts alone. This would turn a patient’s implanted recordings into a running readout of how their network responds to treatment.

## Limitations

We propose that 1) polyspikes and 2) the aperiodic (1/f) slope of the power spectrum provide a fast and interpretable method for identifying sites of excitability that provide putative nodes of epileptic networks in human patients. However, polyspikes are an untested feature for this work, and our own preliminary data only provide a correlation between polyspike duration and amount of cortex recruited. Further, the aperiodic slope carries various analytical confounds. In particular, the slope does not account for power spectral changes that will vary over time in a complex dynamical system (Scott, 2021). Indeed, multiple steps occur during seizure onset, such as varying synchronization and desynchronization (Gorshkov and Ombao, 2024), which can vary across human patients (Jiruska et al., 2013; Schindler et al., 2007) and likely even between individual seizures. We thus emphasize that spectral measures, such as a simple aperiodic slope on a chosen time window, should be viewed as complementary to synchronization-based approaches. Similarly, to better understand the mechanisms of polyspikes, future studies should directly test whether recruited network sites exhibit increases in coupling strength (by synchronization-based methods (Gorshkov and Ombao, 2024)) coinciding with polyspike onsets. While stimulation periods were excluded from the iEEG recording by Feng et al., our RNS analyses reveal a change in aperiodic slope immediately following a stimulation period which could have been biased by lingering effects of stimulation such as a decay of evoked polarization. We also note that our 4-Aminopyridine seizures differ significantly in mechanism from seizures in human focal epilepsy. Finally, we note that our initial results and perspective here come from specific brain networks and regions sampled during focal cortical epilepsy, while great heterogeneity exists in human epilepsies and seizure networks (Zaveri et al., 2026; Zaveri et al., 2020). All of these limitations are clear next-steps for future mechanistic analyses, however, we are optimistic that polyspike and spectral iEEG features can enhance our current ability to identify epileptic networks.

## Conclusion

The seizure onset zone has long been the primary target of epilepsy treatment. However, persistent seizures after apparently successful localization and resection suggest that, in many patients, the seizure onset zone is only one node within a broader epileptic network. We argue that identifying where seizures begin is not adequate. Rather, improving outcomes will require identifying which regions are most excitable, how pathological activity propagates through the network, and which nodes amplify or constrain seizure spread. In this perspective, we propose network excitability mapping as a framework for addressing this problem. We suggest that the aperiodic slope and polyspike structure capture local excitability and network recruitment, two complementary dimensions of epileptic networks. Evidence from electrophysiology, calcium imaging, and computational studies supports the possibility that these measures provide information that is not contained in conventional markers of epileptogenic tissue alone. Importantly, both features can be extracted from recordings that are already routinely obtained in experimental and clinical settings. While substantial validation in human epilepsy remains necessary, these approaches offer a practical path toward constructing patient-specific maps of epileptic network function. By shifting the focus from localization alone to functional characterization of distributed seizure networks, such maps may ultimately improve the selection of surgical targets, guide neuromodulation strategies, and offer more precise therapies for patients with epilepsy.

## Data Availability

The original contributions presented in the study are included in the article/supplementary material, further inquiries can be directed to the corresponding author.
